# Supplementary material for: Fungal–bacterial interaction selects for quorum sensing mutants with increased production of natural antifungal compounds
Source: Commun Biol. 2020 Nov 12;3:670. doi: 10.1038/s42003-020-01342-0 (PMC7661731; doi:10.1038/s42003-020-01342-0)
Supplement: Supplementary file 1 — Supplementary Information [file 42003_2020_1342_MOESM1_ESM.pdf]

**Supplementary Information**

# Fungal-bacterial interaction selects for quorum sensing mutants with increased production of natural antifungal compounds

Andrea G. Albarracín Orio[1,2,3,4]*, Daniel Petras[4,5‡], Romina A. Tobares[2,3‡], Alexander A. Aksenov[4], Mingxum Wang[4], Florencia Juncosa[1], Pamela Sayago[1], Alejandro J. Moyano[2,3], Pieter C. Dorrestein[4]* and Andrea M. Smania[2,3]*

[1]IRNASUS, Universidad Católica de Córdoba, CONICET, Facultad de Ciencias Agropecuarias. Av. Armada Argentina 3555 (X5016DHK) Córdoba, Argentina.
[2]Universidad Nacional de Córdoba. Facultad de Ciencias Químicas. Departamento de Química Biológica Ranwel Caputto. Córdoba, Argentina.
[3]CONICET. Universidad Nacional de Córdoba. Centro de Investigaciones en Química Biológica de Córdoba (CIQUIBIC), Córdoba, Argentina.
[4]Collaborative Mass Spectrometry Innovation Center, 9500 Gilman Drive, University of California, San Diego, CA 92093, USA.
[5]Scripps Institution of Oceanography, University of California San Diego. La Jolla, CA 92093, USA.

Corresponding authors: andrea.albarracin@gmail.com (Andrea G. Albarracín Orio); asmania@fcq.unc.edu.ar (Andrea M. Smania); pdorrestein@ucsd.edu (Pieter C. Dorrestein)

‡ These authors contributed equally to this work

**Supplementary file includes:**

Supplementary Figures 1 to 5
Supplementary Tables 1 to 4
Supplementary Methods
Supplementary References

## Supplementary figures and legends

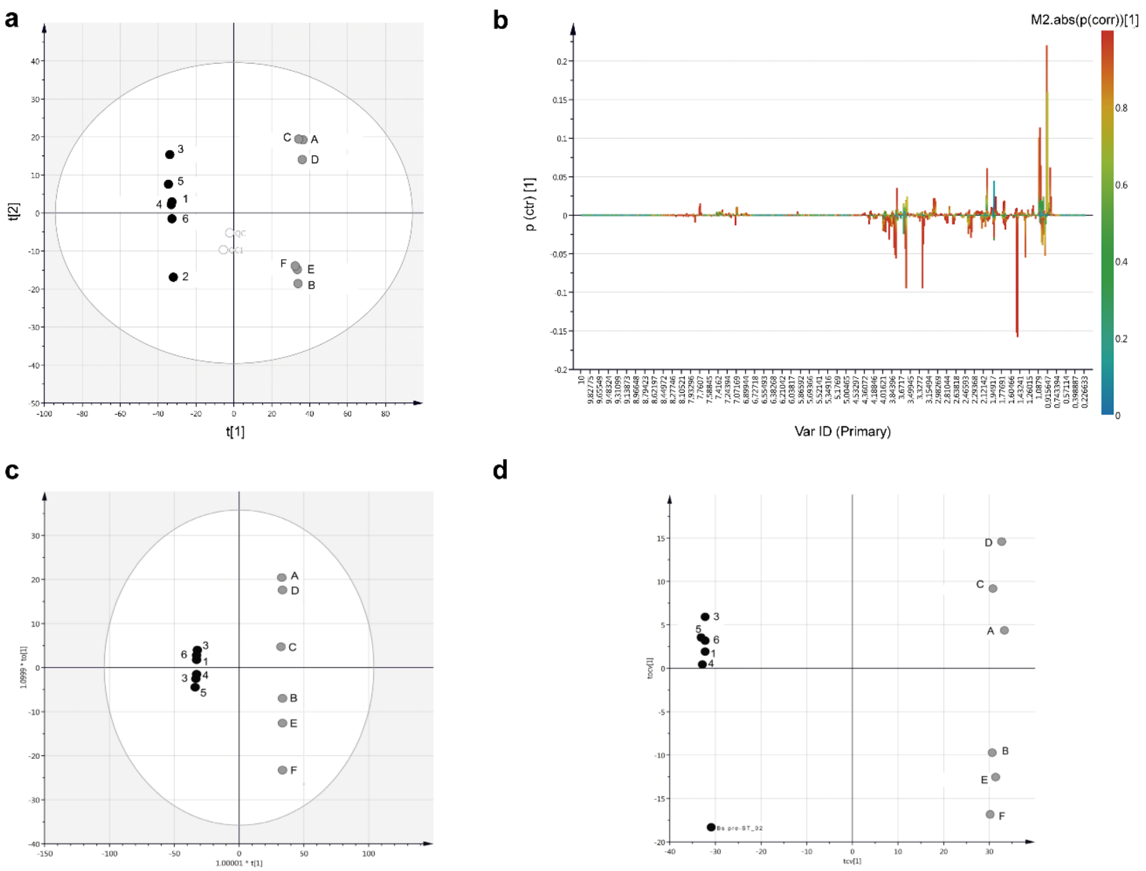


**Supplementary Figure 1. Metabolomics profile variation in *B. subtilis* ALBA01 was observed following *in vitro* interaction with *S. terrestris***

High antifungal activity of cell-free supernatants of post-ST variants correlates with changes in metabolomic profiles of released compounds, since cell-free supernatants of pre- vs. post-ST variants showed detectable differences using untargeted NMR approach. **(a)** Plot of PCA scores shows clear separation of supernatant samples according to their type (black, pre-ST; gray, post-ST) in the first two principal components derived from $^1$H-NMR spectra of *Bacillus*-conditioned media (percent variation in NMR data explained by the model, R2x= 70.4%; percent variation in NMR data predicted by the model from cross-validation, Q2x= 57%, based on 2-components model). White circles: quality control (QC) samples. **(b)** OPLS-DA S-line plots with pairwise comparison of data from NMR spectra from cell-free supernatants of pre- and post-ST. The left y-axis represents p(ctr)[1], the covariance

between a variable and the classification score, which indicates if an increase or decrease of a variable correlates with the classification score. The right y-axis shows p(corr)[1], the correlation coefficient between a variable and the classification score (i.e. the normalized covariance), which provides a linear indication of the correlation strength. Colors are associated with correlation of metabolites characterized from $^{1}$H-NMR data for the class of interest, using the scale on the right with the red color standing for the highest absolute value of the correlation coefficient. Then, chemical shifts (Var ID, x-axis) showing higher correlation values, or red color, represent strongly discriminating variables that separate post-ST from pre-ST samples. **(c)** Plots of OPLS-DA scores and cross-validated scores for pre-ST (black) and post-ST (gray) samples. This analysis was performed to rule out potential bias in sample separation resulting from sample run order. Similar grouping profiles were observed, with two separate clusters for pre- and post-ST samples. **(d)** Orthogonal partial least squares discriminative analysis (OPLS-DA) of data to maximize separation between the groups.

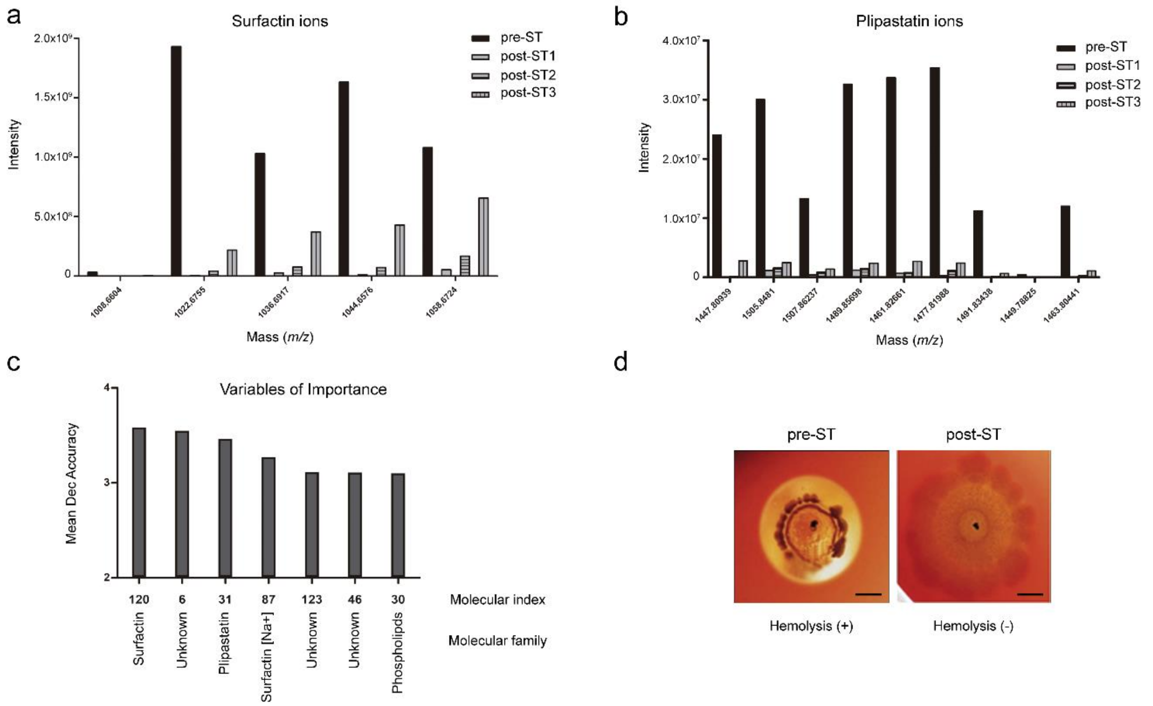


**Supplementary Figure 2. LC-MS/MS-based metabolomic analysis showing chemical signatures that distinguish pre- from post-ST variants**

*B. subtillis* post-ST variants show strongly reduced Intensities of **(a)** surfactin and **(b)** plipastatin ions in whole cell metabolomics analysis. Determinations were performed on three independently obtained post-ST variants (see Methods for details): post-ST1, post-ST2 and post-ST3 (three samples each one) **(c)** Random forest analysis indicated that surfactin and plipastatin are major variables determining such separation. **(d)** Loss of hemolytic activity in post-ST variants. Scale bar: 0.2 cm.

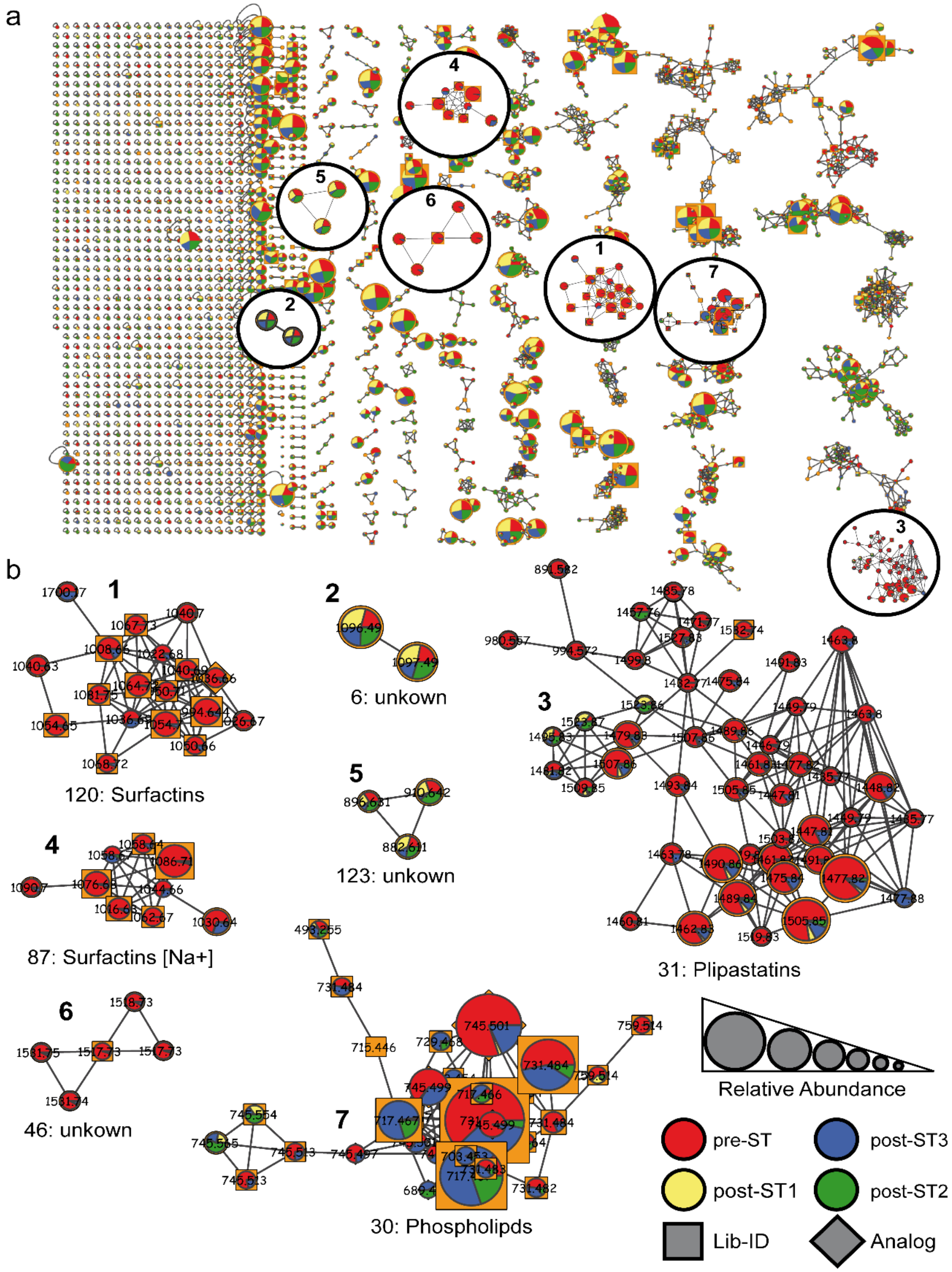
a
b
1
2
3
4
5
6
7
120: Surfactins
6: unkown
87: Surfactins [Na+]
123: unkown
31: Plipastatins
46: unkown
30: Phospholipds
Relative Abundance
pre-ST
post-ST3
post-ST1
post-ST2
Lib-ID
Analog

**Supplementary Figure 3. Molecular networks of MS/MS spectra**

**(a**) Global network of all MS/MS spectra (nodes), connected (edges) on the basis of spectral similarity. **(b)** Magnified images of specific subnetworks (molecular families), which correspond to variables of importance that separate groups according to random forest analysis. Pie charts inside the nodes indicate relative feature abundance (averaged XICs, normalized to TIC of particular groups) between groups. Node size indicates relative feature abundance of all samples (averaged XICs, normalized to TIC of all samples).

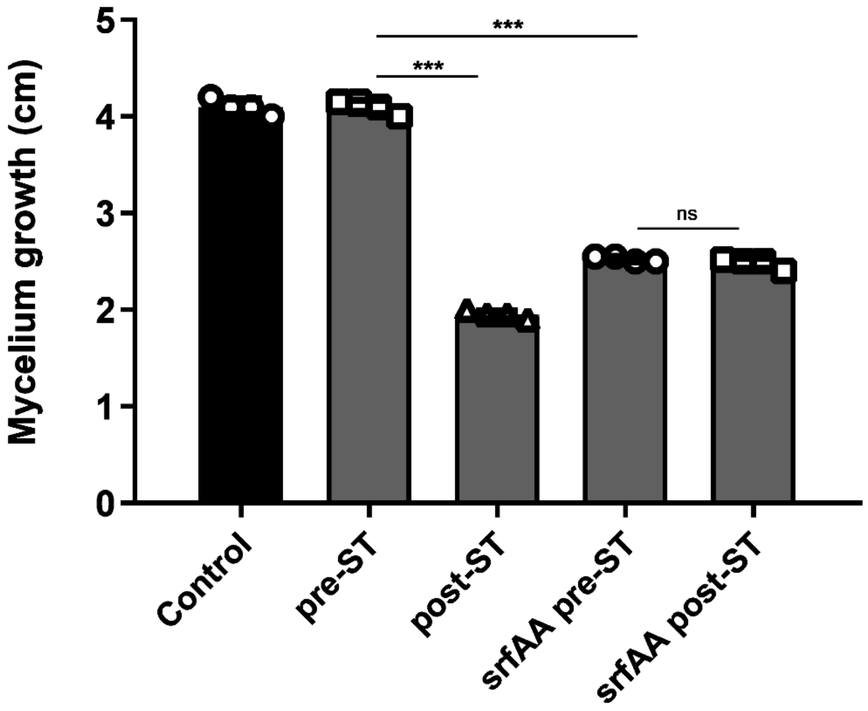


**Supplementary Figure 4. Elimination of surfactin production induces antifungal activity**

Antifungal activities of cell-free supernatants of Bs ALBA01 pre- and post-ST and of its Bs *srfAA* mutant were assayed as described in the text. Cell-free supernatants of surfactin-defective mutant Bs *srfAA* and of Bs ALBA01 post-ST show clear antifungal activity. No changes in the antifungal activity of Bs *srfAA* were observed before (*srfAA* pre-ST) or after (*srfAA* post-ST) interaction with the fungus. Data shown are mean values of mycelial growth from four independent replicate experiments. ***, significant difference between values ($p< 0.0001$, Tukey's Multiple Comparison Test). ns, no significant difference.

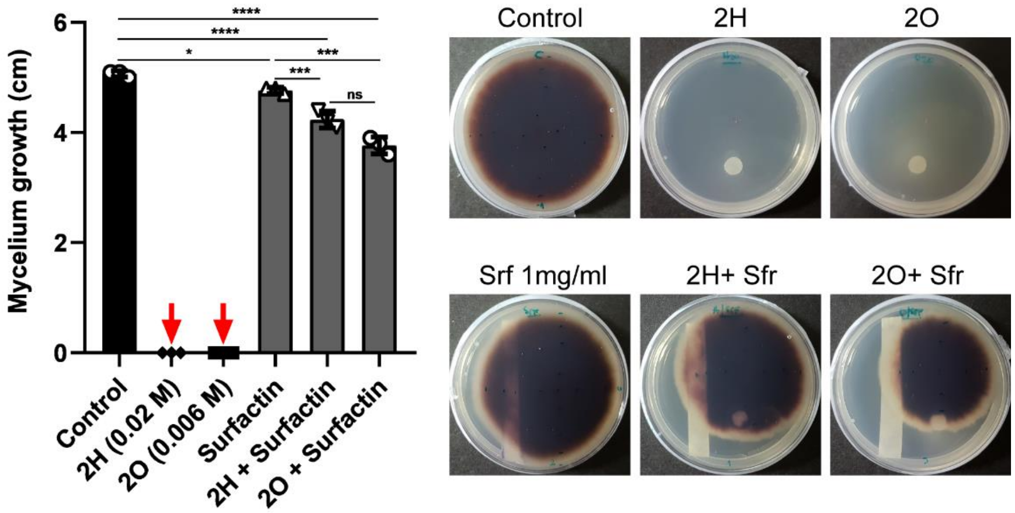


**Supplementary Figure 5. Surfactin interfere with the anti-*S. terrestris* activity of ketones 2-heptanone and 2-octanone**

Suppressor effect of surfactin (1mg/ml imbibed in a filter paper strip with) on the antifungal activity of 2H 0.02 M and 2O 0.006 M, 14 days after inoculation of *S. terrestris*. Data shown are mean values of mycelial growth from three independent replicate experiments; red arrow indicates lethal concentrations of 2-ketones. Statistically significant differences at $p < 0.0001$, $p < 0.001$ and $p < 0.05$ are identified by ***, ** and *, respectively (one-way ANOVA followed by Tukey's Multiple Comparison Test). ns, no significant difference.

## Supplementary Tables

**Supplementary Table 1.** Comparative whole-genome sequencing analysis of independently obtained post-ST variants, and whole-genome sequencing of pools of individuals (Pool-seq), revealed mutations in *comQXPA* coding regions. The +1$A_{415}$ *comA* insertional mutation was found along with three new mutations: two nonsense substitutions (T215A and C601T) and one 5-nucleotide insertion at position 299. The T215A mutation generated a premature stop codon at position 126 of ComA protein, while the C601T and the 5-nucleotide insertion generated stop codons at 201 and 78, respectively. Two new mutations (both insertions) in *comP* were observed: GC insertion at gene position 1517 generated a truncated version of ComP protein with stop codon at position 512, and AT insertion generated a stop codon at ComP position 232.

| Variant | Scaffold/position | Gene | Nucleotide substitution | Description |
|---|---|---|---|---|
| Post-ST 1 | 1/69978 | *comA* | Ins A 415 | Transcriptional regulatory protein ComA |
| Post-ST 2 | 1/69978 | *comA* | Ins A 415 | Transcriptional regulatory protein ComA |
| Post-ST 2 | 2/165291 | *yhfV* | ΔA 1089 | Methyl-accepting chemotaxis protein |
| Post-ST 2 | 3/371952 | *spo0A* | G289C | Sporulation two-component response regulator |
| Post-ST 3 | 1/71309 | *comP* | Δ632-731 | Two-component sensor kinase ComP |
| Pool-seq | 1/69979 | *comA* | C601T | Transcriptional regulatory protein ComA |
| Pool-seq | 1/69978 | *comA* | Ins A 415 | |
| Pool-seq | 1/70101 | *comA* | Ins CTGGG 299 | |
| Pool-seq | 1/70185 | *comA* | T215A | |
| Pool-seq | 1/71264 | *comP* | Ins CG 1517 | Two-component sensor kinase ComP |
| Pool-seq | 1/71919 | *comP* | Ins AT 863 | |

**Supplementary Table 2.** Bacterial and fungal strains used in this study.

| **Strain** | **Characteristics** | **Source** |
|---|---|---|
| *Escherichia coli* DH5α | F-Φ80dlacZ ΔM12 minirecA | Our lab |
| *Bacillus subtilis* ALBA01 | Isolate from onion rhizosphere | Our lab |
| *B. subtilis* pre-ST | *B. subtilis* ALBA01 before co-culture with *S. terrestris* | This study |
| *B. subtilis* post-ST | Variants of *B. subtilis* ALBA01 obtained after co-culture with *S. terrestris* | This study |
| *srfAA* | *B. subtilis* ALBA01 surfactin-defective mutant | This study |
| *srfAA* pre-ST | Surfactin-defective mutant before co-culture with *S. terrestris* | This study |
| *srfAA* post-ST | Surfactin-defective mutant after co-culture with *S. terrestris* | This study |
| *B. subtilis NCBI 3610 srf-deficient* | *B. subtilis* NCBI 3610 *srfAA::mls* | Straight *et al.*, 2006[1] |
| *Setophoma terrestris* PH06 | Fungal strain isolated from onion rhizosphere | Our lab |

**Supplementary Table 3.** Oligonucleotide primers used in this study.

| Primer | Sequence (5' – 3') | Source |
|---|---|---|
| Fsrfa_nul | CGCGGATCCTGACACGATGTTCAGCCTTC | Modified from[2] |
| Rsrfa_nul | GCGGAATTCCAAAACGGTTTCCTTCGGTA | Modified from[2] |
| FcomA_map | TCAAGCAGCATGATTTCTCG | This study |
| RcomA_map | GTCCGTGAACCGACATTCAG | This study |
| FcomP_map | CGATACGTTTGTATAAAAAGCCAAA | This study |
| RcomP_map | TGTGGATTTTATTTTGAGCAGGT | This study |

**Supplementary Table 4.** MZmine2/ADAP settings used for feature finding.

| | |
|---|---|
| **Mass Detection Module** | |
| Mass Detection | Centroid, Noise level 500 |
| Wavelet transform | Wavelet, Noise level 100 |
| Scale level | 5 |
| Wavelet window size (%) | 0.3 |
| **ADAP Chromatogram Builder Module** | |
| Min group size in # of scans | 5.0 |
| Group intensity threshold | 1000.0 |
| Min highest intensity | 1000.0 |
| m/z tolerance | 0.01 Da/20 ppm |
| **Smooting Module** | |
| Filter width | 25 |
| **Deconvolution Module** | |
| Deconvolution | Savitzky-Golay |
| Min peak height | 5000.0 |
| Peak duration range (min) | 0.01-0.2 |
| Derivative threshold level | 0.1 |
| **ADAP3 Decomposition Module** | |
| Min cluster distance (min) | 0.001 |
| Min cluster size | 8 |
| Min cluster intensity | 50000.0 |
| Find shared peaks | False |
| Min edge-to-height ratio | 0.2 |
| Min delta-to-height ratio | 0.2 |
| Min sharpness | 10.0 |
| Shape-similarity tolerance (0..90) | 80.0 |
| Choice of Model Peak based on | Shaprness |
| **Join Aligner Module** | |
| m/z tolerance | 0.01 Da/20 ppm |
| Weight for m/z | 50.0 |
| Retention time tolerance | 0.05 |
| Weight for RT | 0.05 |
| Require same charge state | False |
| Require same ID | False |
| Isotope m/z tolerance | 0.001-5 |
| **Peak Finder Module** | |
| Intensity tolerance | 0.008 |
| m/z tolerance | 0.01 Da/20 ppm |
| Retention time tolerance | 0.05 |
| RT correction | False |

## Supplementary methods

### Biofilm formation, colony morphology, swarming motility and hemolytic activity

For pellicle formation, starting cultures of each strain were grown in 5 ml LB (10 g/L tryptone, 5 g/L yeast extract, 10 g/L NaCl) for 8 h at 37°C with agitation. Three µl of the starting culture were used to inoculate 3 ml LBGM (LB supplemented with 1% glycerol (v/v) and 100 µl $MnSO_4$) in 96-well microtiter plates and in glass tubes, and incubation without agitation was performed at 30°C. For quantification of biofilm formation, unattached cells and media were remove from tubes and/or 96-well microtiter plates and biofilms were stained with 0.1% crystal violet (w/v) for 15 min. Tubes and/or wells were washed twice with 0.1 % NaCl (w/v) and the stained biofilms were solubilized in 125 µl of 30% acetic acid (v/v). Quantification of biofilm production was finally assessed by measuring optical density at 595 nm using 30% acetic acid (v/v) in water as blank. To evaluate colony morphology, 2 µl of the starting culture were spotted onto the surface of LB and LBGM 1.2% agar (w/v) plates, incubated 3-5 days at 30°C, and colonies were analyzed and photographed. To evaluate swarming motility, cells were collected from an overnight colony with a sterile toothpick and inoculated in the center of LB plates with 0.7% agar (w/v). Plates were incubated at 37°C and evaluated for colony spread as a function of time. The capacity of *B. subtilis* pre-ST and post-ST variants to lyse red blood cells was assessed by inoculating 3 µl of starting cultures of each strain, obtained as described above, on 5% blood (v/v) 1.5 % (w/v) agar plates. The test was considered positive when a clear halo was observed around the colonies after an incubation of 18 h at 37°C.

### Obtaining of bacterial cell-free supernatants and fungal inhibition assay

*B. subtilis* pre-ST and post-ST variants were grown in LB broth and incubated for 16–18 h at 30 °C. The cell-free supernatant of each variant was obtained by centrifugation of the

bacterial suspension (10,000 rpm, 10 min) and subsequent filtration (0.2 µm pore size filter). 100 µl of supernatant were plated in PDA and allowed complete diffusion before inoculating a 5 mm diameter agar disk with mycelium of *S. terrestris*. Plates were incubated at 28 °C for 7 days. PDA plates containing only fungal mycelium were used as controls.

**Electron scanning microscopy of *B. subtilis* variants**

*B. subtilis* pre-ST and post-ST variants were exponentially grown at 30 °C in LB broth, and samples for electron microscopy were then collected, centrifuged, and fixed with 4 % formaldehyde-2 % formalin in 0.1 M cacodylate buffer for 1 h at room temperature. An additional fixation with 1% osmium tetroxide in cacodylate buffer was carried out for 1 h at room temperature. These fixed cells were dehydrated using an increasing concentration of acetone, and embedded in polymerized Araldite at 60°C for 48 hours. Thin sections were obtained using a JEOL JUM-7 microtome equipped with a glass or gem grade diamond knife, and microphotography was performed with a Zeiss LEO 906E microscope.

**MS/MS data and featured-based molecular networking analysis**

Following LC-MS/MS data acquisition, raw spectra were converted to .mzXML file format using MSConvert tool (ProteoWizard). MS1 and MS/MS feature extraction was performed with MZmine v. 2.30[3]. Intensity thresholds 1E5 and 1E3 were used for MS1 and MS/MS spectra, respectively. For MS1 chromatogram building, mass accuracy 10 ppm and minimum peak intensity 5E5 were set. Extracted Ion Chromatograms (XICs) were deconvoluted using baseline cutoff at intensity 1E5. After deconvolution, MS1 features were matched to MS/MS spectra within 0.02 m/z and 0.2 min retention time windows. Isotope peaks were grouped, and features from different samples were aligned with mass tolerance 10 ppm and retention time tolerance 0.1 min. MS1 features without assigned MS2 features were filtered out of the resulting matrix, as were features that did not contain isotope peaks

or did not occur in at least three samples. After filtering, gaps in the feature matrix were filled with relaxed retention time tolerance 0.2 min and mass tolerance 10 ppm. The feature table was exported as .csv file and corresponding MS/MS spectra as .mgf. file. Contaminate features observed in Blank samples were filtered, and only those with relative abundance ratio blank to average in the samples <50% were considered for further analysis.

For spectra networking and spectrum library matching, the .mgf file was uploaded to GNPS (gnps.ucsd.edu)[4,5]. For networking, minimum cosine score to define a correlation between spectra was set to 0.7, Precursor Ion Mass Tolerance to 0.01 Da, Fragment Ion Mass Tolerance to 0.01 Da, Minimum Matched Fragment Ions to 4, Minimum Cluster Size to 1 (MS Cluster off), and Library Search Minimum Matched Peaks to 4. When Analog Search was performed, Cosine Score Threshold was 0.7 and Maximum Analog Search Mass Difference was 100. Molecular networks were visualized using software program Cytoscape v. 3.4[6], and node information was matched with MS1 feature table. Tridimensional PCoA plots of MS1 data were generated by in-house tool ClusterApp using Bray-Curtis distance metric. Resulting scatter plots were visualized on EMPeror. Random forest classification was performed in R.

**$^{1}H$-NMR spectroscopy-based metabolic profiling of cell-free supernatants of *B. subtilis* pre- and post-ST variants**

$^{1}H$-NMR spectroscopy and multivariate data analysis were performed at PLABEM (Plataforma Argentina de Biología Estructural y Metabolómica; Rosario, Argentina). Samples were prepared for $^{1}H$-NMR as described in[7]. Briefly, 540 µl of the supernatant were mixed with 60 µl phosphate buffer (pH 7.4) containing sodium 3-trimethylsilyl-(2,2,3,3-$^{2}H_4$)-1-propionate (TSP) in $D_2O$ (final concentration 0.1 mg/mL). TSP acts as internal chemical shift reference ($\delta$= 0.0), while $D_2O$ provides lock signal for the spectrometer. Samples were stood for 10 min, then centrifuged at 4000 rpm for 10 min to remove any precipitates. 500 µl

of centrifuged solution were transferred to NMR tube. A pooled quality control sample (QC (2)) was prepared by mixing equal volumes (100 µL) of all 12 samples.

Spectra were obtained at 300 K using an Avance 600 MHz NMR spectrometer (Bruker BioSpin; Rheinstetten, Germany) equipped with 5-mm TXI probe. One-dimensional $^1$H-NMR spectra of conditioned culture media were acquired using standard 1-D NOESY pulse sequence (noesygppr1d) with water presaturation[8]. Mixing time was set to 10 ms, data acquisition period to 2.73 s, and relaxation delay to 4 µs. $^1$H-NMR spectra were acquired using 4 dummy scans and 32 scans, with 64K time domain points and spectral window 20 ppm. FIDs were multiplied by an exponential weighting function corresponding to line broadening 0.3 Hz.

Spectroscopic data was processed by MATLAB v. R2015b (MathWorks Inc.; U.S.). Spectra were referenced to TSP at 0.0 ppm, with baseline correction and phasing of spectra performed using in-house software (provided by T. Ebbels and H. Keun, Imperial College, UK). Each spectrum was reduced to a series of integrated regions of equal width (0.04 ppm, standard bucket width). Spectral regions containing no metabolite signals and TSP signal <0.2 ppm, and the interval containing the water signal (between 4.9 and 4.6 ppm) were excluded. Each spectrum was then normalized by probabilistic quotient method[9].

Pre-processed $^1$H-NMR spectral data were imported to SIMCA (v. 14.1, Umetrics AB; Umeå, Sweden) for multivariate data analysis. Principal Component Analysis (PCA) was performed using the Pareto-scaled NMR dataset. Orthogonal partial least squares discriminative analysis (OPLS-DA) was performed to maximize separation between treatment groups. S-line plots (tailored S-plots)[10] useful for NMR data analysis) were generated to visualize differences between classes in OPLS-DA models. Full cross validation (CV) was performed to ensure valid and reliable models and to avoid overfitting[11].

## Supplementary References


1. Straight PD, Willey JM, Kolter R. Interactions between *Streptomyces coelicolor* and *Bacillus subtilis*: Role of Surfactants in Raising Aerial Structures. *Journal of Bacteriology* **188**, 4918-4925 (2006).

2. Bais HP, Fall R, Vivanco JM. Biocontrol of *Bacillus subtilis* against infection of *Arabidopsis* roots by *Pseudomonas syringae* is facilitated by biofilm formation and surfactin production. *Plant Physiology* **134**, 307-319 (2004).

3. Pluskal T, Castillo S, Villar-Briones A, Orešič M. MZmine 2: Modular framework for processing, visualizing, and analyzing mass spectrometry-based molecular profile data. *BMC Bioinformatics* **11**, 395 (2010).

4. Wang M*, et al.* Sharing and community curation of mass spectrometry data with Global Natural Products Social Molecular Networking. *Nature Biotechnology* **34**, 828 (2016).

5. Kjeldgaard B, Listian SA, Ramaswamhi V, Richter A, Kiesewalter HT, Kovács ÁT. Fungal hyphae colonization by *Bacillus subtilis* relies on biofilm matrix components. *Biofilm* **1**, 100007 (2019).

6. Shannon P*, et al.* Cytoscape: A software environment for integrated models of biomolecular interaction networks. *Genome Research* **13**, 2498-2504 (2003).

7. Dona AC*, et al.* Precision high-throughput proton NMR spectroscopy of human urine, serum, and plasma for large-scale metabolic phenotyping. *Analytical chemistry* **86**, 9887-9894 (2014).

8. Nicholson JK, Foxall PJ, Spraul M, Farrant RD, Lindon JC. 750 MHz 1H and 1H-13C NMR spectroscopy of human blood plasma. *Analytical Chemistry* **67**, 793-811 (1995).

9. Dieterle F, Ross A, Schlotterbeck G, Senn H. Probabilistic quotient normalization as robust method to account for dilution of complex biological mixtures. Application in 1H NMR metabonomics. *Analytical Chemistry* **78**, 4281-4290 (2006).

10. Wiklund S*, et al.* Visualization of GC/TOF-MS-based metabolomics data for identification of biochemically interesting compounds using OPLS class models. *Analytical Chemistry* **80**, 115-122 (2008).

11. Kjeldahl K, Bro R. Some common misunderstandings in chemometrics. *Journal of Chemometrics* **24**, 558-564 (2010).
