# Supplementary material for: Fungal–bacterial interaction selects for quorum sensing mutants with increased production of natural antifungal compounds
Source: Commun Biol. 2020 Nov 12;3:670. doi: 10.1038/s42003-020-01342-0 (PMC7661731; doi:10.1038/s42003-020-01342-0)
Supplement: Supplementary file 3 — Description of additional supplementary files [file 42003_2020_1342_MOESM3_ESM.docx]

Description of Additional Supplementary Files

Supplementary Data 1 and 2:

Source data underlying plots shown in figures.
