# Supplementary material for: Fungal–bacterial interaction selects for quorum sensing mutants with increased production of natural antifungal compounds
Source: Commun Biol. 2020 Nov 12;3:670. doi: 10.1038/s42003-020-01342-0 (PMC7661731; doi:10.1038/s42003-020-01342-0)
Supplement: Supplementary file 6 — Reporting Summary [file 42003_2020_1342_MOESM6_ESM.pdf]

nature research

Corresponding author(s): Andrea M. Smania, Andrea G. Albarracín Orio and Pieter C. Dorrestein

Last updated by author(s): Jul 14, 2020

Reporting Summary

Nature Research wishes to improve the reproducibility of the work that we publish. This form provides structure for consistency and transparency in reporting. For further information on Nature Research policies, see Authors & Referees and the Editorial Policy Checklist.

Statistics

For all statistical analyses, confirm that the following items are present in the figure legend, table legend, main text, or Methods section.

n/a	Confirmed	
☐	☒	The exact sample size (n) for each experimental group/condition, given as a discrete number and unit of measurement
☐	☒	A statement on whether measurements were taken from distinct samples or whether the same sample was measured repeatedly
☐	☒	The statistical test(s) used AND whether they are one- or two-sided<br>*Only common tests should be described solely by name; describe more complex techniques in the Methods section.*
☒	☐	A description of all covariates tested
☒	☐	A description of any assumptions or corrections, such as tests of normality and adjustment for multiple comparisons
☐	☒	A full description of the statistical parameters including central tendency (e.g. means) or other basic estimates (e.g. regression coefficient) AND variation (e.g. standard deviation) or associated estimates of uncertainty (e.g. confidence intervals)
☐	☒	For null hypothesis testing, the test statistic (e.g. F, t, r) with confidence intervals, effect sizes, degrees of freedom and P value noted<br>*Give P values as exact values whenever suitable.*
☒	☐	For Bayesian analysis, information on the choice of priors and Markov chain Monte Carlo settings
☒	☐	For hierarchical and complex designs, identification of the appropriate level for tests and full reporting of outcomes
☒	☐	Estimates of effect sizes (e.g. Cohen's d, Pearson's r), indicating how they were calculated

Our web collection on statistics for biologists contains articles on many of the points above.

Software and code

Policy information about availability of computer code

Data collection	Genome sequencing data were collected using commercial softwares from Illumina. Mass spectrometry-based metabolomics data were obtained with the commercial software of Thermo Scientific for UHPLC systems and Agilent for GC systems. For 1H-NMR spectroscopy-based metabolic profiling, data were collected with the commercial software of Bruker Avance 600 MHz NMR spectrometer.
Data analysis	Several programs were used throughout this study to analyze the data.<br><br>For whole genome analysis, de novo assembly was performed on the A5 pipeline. For annotation of the genomes, scaffolds were uploaded to Rapid Annotation using Subsystem Technology (RAST) server, and SEED-based method was applied on this server. To map the reads the BWA-MEM tool (v. 0.7.5a-r405) was used. Variants were called using Genome Analysis Toolkit (GATK) HaplotypeCaller. Integrative Genomics Viewer (IGV) was used for manual inspection of variants and read alignments.<br><br>For HPLC-MS/MS analysis, raw spectra were converted to .mzXML file format using MSConvert tool (ProteoWizard). MS1 and MS/MS feature extraction was performed with MZmine v. 2.30. Intensity thresholds 1E5 and 1E3 were used for MS1 and MS/MS spectra, respectively. For MS1 chromatogram building, mass accuracy 10 ppm and minimum peak intensity 5E5 were set. Extracted Ion Chromatograms (XICs) were deconvoluted using baseline cutoff at intensity 1E5. After deconvolution, MS1 features were matched to MS/MS spectra within 0.02 m/z and 0.2 min retention time windows. Isotope peaks were grouped, and features from different samples were aligned with mass tolerance 10 ppm and retention time tolerance 0.1 min. MS1 features without assigned MS2 features were filtered out of the resulting matrix, as were features that did not contain isotope peaks or did not occur in at least three samples. After filtering, gaps in the feature matrix were filled with relaxed retention time tolerance 0.2 min and mass tolerance 10 ppm. The feature table was exported as .csv file and corresponding MS/MS spectra as .mgf file. Contaminate features observed in Blank samples were filtered, and only those with relative abundance ratio blank to average in the samples <50% were considered for further analysis.<br><br>For feature-based molecular networking, the .mgf file was uploaded to GNPS (gnps.ucsd.edu). For networking, minimum cosine score to define a correlation between spectra was set to 0.7, Precursor Ion Mass Tolerance to 0.01 Da, Fragment Ion Mass Tolerance to 0.01 Da, Minimum Matched Fragment Ions to 4, Minimum Cluster Size to 1 (MS Cluster off), and Library Search Minimum Matched Peaks to 4.

When Analog Search was performed, Cosine Score Threshold was 0.7 and Maximum Analog Search Mass Difference was 100. Molecular networks were visualized using software program Cytoscape v. 3.4, and node information was matched with MS1 feature table. Tridimensional PCoA plots of MS1 data were generated by in-house tool ClusterApp using Bray-Curtis distance metric. Resulting scatter plots were visualized on EMPeror. Random forest classification was performed in R.

GC-MS data were processed with MZmine2 (https://bmcbioinformatics.biomedcentral.com/articles/10.1186/1471-2105-11-395) using ADAP algorithm (https://pubs.acs.org/doi/full/10.1021/acs.jproteome.7b00633?src=recsys) deployed on the ProteoSAFE workflow of the GNPS platform (gnps.ucsd.edu). The complete list of parameters is shown in the text in Supplementary Table 4.

Spectroscopic data was processed by MATLAB v. R2015b (MathWorks Inc.; U.S.). Spectra were referenced to TSP at 0.0 ppm, with baseline correction and phasing of spectra performed using in-house software (provided by T. Ebbels and H. Keun, Imperial College, UK). Each spectrum was reduced to a series of integrated regions of equal width (0.04 ppm, standard bucket width). Spectral regions containing no metabolite signals and TSP signal <0.2 ppm, and the interval containing the water signal (between 4.9 and 4.6 ppm) were excluded. Each spectrum was then normalized by probabilistic quotient method. Pre-processed 1H-NMR spectral data were imported to SIMCA (v. 14.1, Umetrics AB; Umeå, Sweden) for multivariate data analysis. Principal Component Analysis (PCA) was performed using the Pareto-scaled NMR dataset.

For manuscripts utilizing custom algorithms or software that are central to the research but not yet described in published literature, software must be made available to editors/reviewers. We strongly encourage code deposition in a community repository (e.g. GitHub). See the Nature Research guidelines for submitting code & software for further information.

Data

Policy information about availability of data

All manuscripts must include a data availability statement. This statement should provide the following information, where applicable:

- Accession codes, unique identifiers, or web links for publicly available datasets
- A list of figures that have associated raw data
- A description of any restrictions on data availability

Nucleotide sequence accession numbers: sequence of Bacillus subtilis ALBA01 assembled genome was deposited in NCBI database (Bioproject PRJNA316980). Genome sequence reads from post-ST variants and Pool-seq analysis were deposited in NCBI database (Bioproject PRJNA480851).
HPLC-MS/MS D data can be found on the Mass spectrometry Interactive Virtual Environment (MassIVE) at https://massive.ucsd.edu/ with the identifier MSV000082081. The GC-MS data are available at the MassIVE depository under ID MSV000083294.

Field-specific reporting

Please select the one below that is the best fit for your research. If you are not sure, read the appropriate sections before making your selection.

☒ Life sciences ☐ Behavioural & social sciences ☐ Ecological, evolutionary & environmental sciences

For a reference copy of the document with all sections, see nature.com/documents/nr-reporting-summary-flat.pdf

Life sciences study design

All studies must disclose on these points even when the disclosure is negative.

Sample size	No sample size calculation was performed. A sufficient number of replicates were taken in order to get statistically confident results. In all the experiments standard deviations were calculated, and Tukey's analysis was done when needed.
Data exclusions	No data were excluded from calculation.
Replication	At least three replicates were performed in all the experiments. In all cases the experiments were successfully replicated.<br>Whole genome sequencing was performed on three Bacillus subtilis post-ST variants (post-ST1, post-ST2, post-ST3) derived independently from Bacillus subtilis ALBA01 (pre-ST ancestor) following interaction with the fungus in co-culture. For Pool-seq sequencing, we used 15 addtional and different post-ST variants which were obtained from independent experiments of interaction between Bacillus subtilis ALBA01 and the fungus grown in co-culture.
Randomization	Randomization was not relevant in this case, since our study is mainly focused on a bacterial-fungal interaction.
Blinding	No blinding was necessary

Reporting for specific materials, systems and methods

We require information from authors about some types of materials, experimental systems and methods used in many studies. Here, indicate whether each material, system or method listed is relevant to your study. If you are not sure if a list item applies to your research, read the appropriate section before selecting a response.

Materials & experimental systems

n/a	Involved in the study
☒	☐ Antibodies
☒	☐ Eukaryotic cell lines
☒	☐ Palaeontology
☒	☐ Animals and other organisms
☒	☐ Human research participants
☒	☐ Clinical data

Methods

n/a	Involved in the study
☒	☐ ChIP-seq
☒	☐ Flow cytometry
☒	☐ MRI-based neuroimaging
